# Introducing the Hungarian Version of the SCREENIVF Tool into the Clinical Routine Screening of Emotional Maladjustment

**DOI:** 10.3390/ijerph191610147

**Published:** 2022-08-16

**Authors:** Viktória Prémusz, Pongrác Ács, József Bódis, Ákos Várnagy, Ágnes Lászik, Alexandra Makai

**Affiliations:** 1 Faculty of Health Sciences, University of Pécs, H-7624 Pécs, Hungary; 2 ELKH-PTE Human Reproduction Scientific Research Group, University of Pécs, H-7624 Pécs, Hungary; 3 National Laboratory on Human Reproduction, University of Pécs, H-7624 Pécs, Hungary; 4 Department of Obstetrics and Gynaecology, Medical School, University of Pécs, H-7624 Pécs, Hungary

**Keywords:** infertility, assisted reproduction, IVF, ART, validation, psychosocial factors, SCREENIVF, clinical routine care, screening, emotional maladjustment

## Abstract

Examining possible psychosocial maladjustments should be an integral part of fertility care. For the early detection of vulnerability, the present study aimed to adapt and test the reliability and validity of the Hungarian version of SCREENIVF against the Fertility Quality of Life Questionnaire (FertiQoL) in a cross-sectional on subfertile women (*n* = 60, age 34.6 ± 5.2 years, BMI 24.2 ± 4.9 kg/m^2^) at a university linked fertility clinic in South-Hungary. A confirmatory factor analysis (CFA) was conducted to investigate the construct validity. For the reliability testing, Cronbach alpha values were calculated. Spearman’s rank correlation tested the criterion validity. Discriminant validity was applied using Mann–Whitney U-test and Kruskal–Wallis test. The Edinburgh Framework and COSMIN checklist were applicable for the analysis using SPSS 27.0; significance was set at *p* < 0.05. The confirmatory factor analysis indicated a good fit; all dimensions were reliable (α ≥ 0.70). Cronbach’s alpha was excellent (0.825–0.904). Strong correlations were found between the total scale (FertiQoL) and anxiety (R = −0.507, *p* < 0.001), depression (R = 0.554, *p* < 0.001), and helplessness cognitions (R = −0.747, *p* < 0.001) and moderate or no correlation with acceptance cognitions (R = 0.317, *p* = 0.015) and social support (R = 0.230, *p* = 0.082). The Hungarian version of SCREENIVF proved a valid and reliable tool to measure psychological maladjustment before ART. A longitudinal, randomized, controlled trial involving the partners could further strengthen the results, which is among our long-term plans.

## 1. Introduction

One of every ten or even one of every six couples is affected by one-year infertility [1,2] in Europe [3,4,5]. Data on the prevalence of infertility varies wildly between studies based on the variability in sampling [6,7,8,9,10,11,12] and the definition of infertility [6,13]. However, there is no doubt about its public health significance and the importance of its clinical management [14]. The need for infertility medical care is notable [7,8,9], although only 56–59% of the couples seek medical help for fertility problems and demand medical service after experiencing a 12-month delay in conception while not using contraception [4,15,16].

Couples often deal with the experience of infertility for several years before medical help-seeking and during the treatments [3,17]. No compelling evidence exists so far that emotional distress (anxiety, depression) affects the outcome of assisted reproduction treatment (ART). However, infertility and the perceived lack of control by patients during ART treatment make the situation itself a long-term stressor. The treatment and particularly a failed treatment, could be emotionally and physically demanding [18,19,20] and may increase perceived distress, anxiety, and depression [21,22] and decrease quality of life [19,23].

The European Society of Human Reproduction and Embryology (ESHRE) recommends that fertility clinics provide routine psychosocial care to patients dealing with infertility and ART [24]. The guideline describes in detail the patients’ preferences and experience regarding the psychosocial care across their treatment (before, during, and after treatment), how this care is associated with their well-being, and how fertility clinic staff can detect and address these needs. “Needs can be behavioral (lifestyle, exercise, nutrition, and compliance), relational (relationship with a partner if there is one, family friends and a larger network, and work), emotional (well-being, e.g., anxiety, depression, quality of life), and cognitive (treatment concerns and knowledge)” [24].

The Polish Psychiatric Association also draws the attention of fertility professionals to psychological susceptibility. Their recommendation for treating affective disorders in women of childbearing age also recommends screening patients with pregnancy loss and unsuccessful IVF with SCREENIVF and applying the tool in clinical practice to identify patients at risk of developing psychopathological symptoms [25].

In Hungary, some studies were conducted about the psychosocial burden [26,27,28,29] and lifestyle habits [29,30,31] of ART patients, but their main focus was on the prognostic value of outcome measures. The psychosocial care of ART patients in Hungary is only sporadically solved; there is no routine screening for early vulnerability detection. Treatment-as-usual (TAU) does not include psychosocial counseling in Hungary. It depends on the participant’s attitude, the attending physician’s awareness, and the clinic’s service portfolio (usually private clinics), whether they recommend using a consultation, mainly based on out-of-pocket payments. 

However, at the start of ART, the risk of emotional maladjustment during or after the treatment could be detected by SCREENIVF [2,32,33,34,35], and additional psychosocial support could be provided for those patients who are identified to be at risk, which screening could be an integral part of TAU.

Therefore, the purpose of this study was the cultural adaptation and validity and reliability testing of SCREENIVF to the Hungarian population. We aimed to develop a Hungarian version equivalent to the original English version, translate and culturally adapt it to the target population, and assess its psychological properties in a cross-sectional study in Hungary.

## 2. Materials and Methods

### 2.1. Translation and Cross-Cultural Adaptation

The translation and adaptation processes of the Hungarian version were carried out through six stages following the Guidelines for the Process of Cross-Cultural Adaption of Self-Report Measures by Beaton [36], with the author’s written consent (© Verhaak CM, Radboud University Medical Center Nijmegen the Netherlands). The translation process from English to Hungarian was based on forward and backward translation. First, the forward translation was done by one informed (medical English expert) and one uninformed independent bilingual translator whose native language was Hungarian. Then the synthesis of the translations was created by the authors. In the third stage, the backward translation and comparison between the Hungarian and English versions were completed by two bilingual translators. One was a professional medical translator and the other was a gynecologist working at the reproduction unit with highly advanced English language certification. The fourth stage included an expert committee discussion, where developers, a methodologist, translators and physicians, a head nurse, and an embryologist of the reproduction unit were invited. Each version provided written reports, and the committee reviewed any discrepancies before reaching a final consensus. In the last stage of the translation and adaptation process, the pre-final version of the instrument was piloted on ten ART patients with a special focus on the involvement of respondents with lower levels of education to assure comprehension and ease of administration. Only minor changes were made after the pilot testing. The final Hungarian version of SCREENIVF was used for the second phase of this study. This included an assessment of the tool’s psychometric properties on a consecutive sample of 60 subfertile women and measuring their emotional maladjustment.

### 2.2. Selection and Description of Participants

A cross-sectional study with the purpose of validation was nested in a cohort study to analyze the effect of psychosocial and lifestyle factors—with special regard to self-reported and objective measures of pre-treatment habitual physical activity—on the course and primary and secondary outcome measures of ART. The specific purpose of the main study reasoned to strict inclusion criteria. To examine the effect of lifestyle and psychosocial factors, we intended to allocate a study population as homogeneous as possible and exclude factors that fundamentally affect the outcome of ART or physical activity, such as age, metabolic, vascular, or receptivity-related factors. Therefore, the involvement of the participants took place as follows.

According to the date of the fertility consultation, 75 female patients with infertility diagnoses and indicated for fertility treatment (Pre-ART/ART) were consecutively invited to a cross-sectional, observational cohort study at the Assisted Reproduction Unit, Department of Obstetrics and Gynecology, University of Pécs, Hungary. Inclusion criteria were BMI ≥ 18 kg/m^2^ and ≤38 kg/m^2^, 18 to 40 years of age, and no metabolic and vascular diseases including diabetes mellitus, fatty liver diseases and atherosclerosis, severe endometriosis and/or adenomyosis, or any mental disorders. Even though only women were included in our study, the instrument itself is suitable for assessing both members of couples and even for assessing patients diagnosed with any co-morbidity for whom ART treatment has been prescribed. 

The aim of the study was explained to each participant, and written informed consent was obtained before the research began by trained researchers. Data collection was carried out during the routine examination on the 3rd day of the unstimulated cycles. Then, 62 women filled out self-administered questionnaires in paper-pencil form between December 2018 and June 2019 at home. They returned the questionnaires on the 21st day by the second consultation, but two of the returned questionnaires should be excluded due to the high number of missing answers.

The major socio-demographic characteristics of the study population (*n* = 60) could be considered favorable compared to the average Hungarian population [37]. Only a minor proportion of the participants could be characterized by low educational degree (10.0%) and economic status (3.3%), unemployment (5.0%), or rural residence (25.0%). Each participant was of reproductive age (34.6 ± 5.2 years), lived in a relationship, and tried to conceive for 60 months on average (59.0 ± 38.4 months). Most received In-Vitro Fertilization/Intracytoplasmic Sperm Injection (IVF/ICSI).

IVF/ICSI (82.3%) due to female indications (75.6%). 

In general, participants rated their health particularly good or excellent (76.7%) and self-reported a health-conscious lifestyle regarding diet, tobacco use, exercise, and quality of sleep; 70.0% had normal weight (BMI 18.5–24.9 kg/m^2^) (Table 1).

### 2.3. Validity and Reliability Process 

The Edinburgh Framework for validity and reliability [38] and the COSMIN checklist [39,40] were applicable for the analysis. 

### 2.4. Assessment Scales

Socio-demographic characteristics were obtained using questions regarding gender, age, education, income, marital status, relationship duration, and area of residence. Health status was also examined. BMI, self-rated health, diet, smoking, and exercise habits were asked. Self-reported fertility information included duration of the partnership and infertility, parity, number of previous treatments, and current stage of treatment.

The assessment of psychological maladjustment was conducted using Version 2.0 of the SCREENIVF tool [2,32]. This multidimensional questionnaire contains 34 questions and assesses anxiety (10 items from the short version of the Spielberger State and Trait Anxiety Inventory; the validated Hungarian form of the questions was used [41,42]), depression (7 items from the Beck Depression Inventory, validated in Hungarian as well [43,44]), social support (5 items derived from the Inventory of Social Involvement [45]), and helplessness and acceptance cognitions (6 + 6 items from the Illness Cognition Questionnaire for IVF Patients [33,46]. Individuals at risk were classified following the screening concept of Verhaak et al. [32]. Cut-off points are presented in Table 2. An at-risk score can be calculated when one or more of the five dimensions shows a clinically relevant problem.

Fertility-related quality of life (QoL) was measured with the Hungarian version of the Fertility Quality of Life Questionnaire (FertiQoL) [28,30,47,48]. FertiQoL is an internationally validated instrument to measure QoL in individuals experiencing fertility problems in diverse life areas. The core module describes general health, self-perceptions, emotions, partnership, family and social relationships, work life, and future life plans (26 items) along four domains (emotional, mind-body, relational, and social scale). The optional FertiQoL Treatment module (10 items) assesses the environment and tolerability of fertility treatment [23,47].

Social support was also evaluated with an independent tool adopted by the Hungarian 3-item version of Caldwell’s Social Support Questionnaire (SSQ) [49]. The questionnaire asks whom the respondent can expect to help in a difficult life situation. The value of support was scored 0–3. We worked with the total score of the questionnaire and examined how much social support the respondent could expect based on his/her perception.

The validated Hungarian short-form of the Beck Depression Inventory (BDI-13) was applied for reporting respondents’ mental health status [50,51,52,53]. BDI is widely used to measure the intensity of depression in the general population and infertile patients [54,55]. The tool asks how the respondent has been feeling in the last week. Answers range in intensity, and each question has a set of at least four possible responses. For the outcome index of depression, a total score is computed.

### 2.5. Data Analysis

Data were entered in Microsoft Excel and analyzed using IBM SPSS 27.0 program. Descriptive analysis was carried out. The data were expressed by the mean and standard deviation (SD), median and interquartile range (IQR), and frequency (%).

The normality of the data was tested using the Kolmogorov–Smirnov test (data was considered normally distributed if *p* < 0.05). Construct validity and reliability were investigated to control psychometric properties. The confirmatory factor analysis (CFA) was performed using AMOS, version 24.0, to test the structure of the tool. 

The model was a first-order model with five latent variables that correspond to the five risk factors for emotional maladjustment.

To assess model fit, commonly used parameters were analyzed: χ^2^, the comparative fit index (CFI), and the root-mean-square error of approximation (RMSEA) [56]. A model is considered to have a very good fit if the χ^2^ value is non-significant (*p* > 0.05), the CFI is 0.95, and the RMSEA is 0.06 [57]. 

The Cronbach Alpha value was calculated for all tool dimensions to measure the reliability.

Associations between the five SCREENIVF risk factors and between the risk factors and the QoL domains were investigated. The convergent validity between the questionnaires (SCREENIVF and FertiQoL, Caldwell SSQ, BDI-13) was examined using Spearman’s rank correlation [58]. Discriminant validity was applied using the Mann–Whitney U-test and Kruskal–Wallis test to test the difference between dimensions of psychological maladjustment based on the Hungarian version of SCREENIVF and factors with possible psychological impact.

A confidence interval of 95% was applied, and a *p*-value of <0.05 was considered statistically significant.

## 3. Results

### 3.1. Psychological Characteristics of the Sample 

There were 50.00% of the women at risk at least in two dimensions of SCREENIVF. The ratio of respondents at risk concerning the original cut-off values was the highest (56.9%) in the dimension of Anxiety and lowest in Acceptance cognitions (20.7%) (Table 2).

For measuring the level of infertility-related QoL, the FertiQoL was applied. In the four core subscales of the questionnaire, we found lower QoL on the Emotional Scale (57.47 ± 18.47) and the Mind-Body Scale (58.62 ± 19.27), mediate QoL on the Social Scale (63.58 ± 17.50) and moderately better values by the Relational Scale (77.59 ± 18.30). Subscales were summarized, and the Core Scale was 64.31 ± 13.90. Mediate QoL was also evaluated related to the Environment (66.24 ± 21.38) and Tolerability (62.50 ± 26.41) of the treatment. 73.3% of the study population self-reported normal mood state [4.84 (SD = 4.88)] with BDI-13, SSQ scored on average 13.14 (SD = 1.89) (Table 3).

### 3.2. Validity and Reliability of the Hungarian SCREENIVF Tool

#### 3.2.1. Construct Validity CFA

The confirmatory factor analysis was calculated using IBM SPSS AMOS 24.0. The model fit statistics showed a good fit based on CFI and RMSEA. (CFI Ľ 0.955; RMSEA Ľ 0.047 [90% confidence interval 0.016–0.067]. The correlations between the factors were between −0.374–0.745.

Correlations between the risk factors on psychological maladjustment measured with the dimensions of SCREENIVF have also been investigated. The closest relationship was found between depression, anxiety, and helplessness. The correlation coefficient between depression and anxiety and between helplessness and anxiety or helplessness and depression were strong (R > 0.50), while a moderate relationship (0.30 < R < 0.50) was found between Social Support, Anxiety, and Depression. Acceptance cognitions were also negatively correlated with Depression and Helplessness cognitions (Table 4).

#### 3.2.2. Reliability of the SCREENIVF Dimensions

The Cronbach’s alpha value for each dimension was determined, and the correlation of all dimension-subscales exceeded the value of 0.700. All dimensions adequately represent the concept that the tool measures [59]. Cronbach’s alpha varied from 0.825 to 0.904, reliability of all dimensions was excellent (depression Cronbach’s α = 0.825, anxiety Cronbach’s α = 0.850, acceptance Cronbach’s α = 0.859, helplessness Cronbach’s α = 0.863, social support Cronbach’s α = 0.904).

#### 3.2.3. Criterion Validity

Criterion validity correlation coefficients of the SCREENIVF and FertiQoL, BDI-13, and Caldwell SSQ are shown in Table 5. A negative correlation was found between quality-of-life scales, depression, and helplessness cognition dimensions and positive with acceptance cognitions dimension.

Helplessness cognitions significantly correlated with all subscales of FertiQoL, SSQ, and BDI-13, as anxiety and depression as well, except for the environment scale. As expected, acceptance cognitions were negatively correlated with BDI-13 (R = −0.369, *p* = 0.004) and positively correlated with the Emotional, Mind-Body, Social, and Tolerability Scale of FertiQoL.

We were able to prove fewer relationships using the social support dimension of SCREENIVF: significant correlations were found only with SSQ (R = 0.306, *p* = 0.019), BDI-13 (R = −0.468, *p* **<** 0.001), and relational subscale of FertiQoL (R = 0.341, *p* = 0.009) and against the assumptions no correlation could be described with the Social Scale of FertiQoL (R = 0.192, *p* = 0.149). Strong correlations were found between the total scale of FertiQoL and anxiety (R = −0.507, *p* < 0.001), depression (R = −0.554, *p* < 0.001), and helplessness cognitions (R = −0.747, *p* < 0.001) and moderate correlation with acceptance cognitions (R = 0.317, *p* = 0.015) (Table 5).

#### 3.2.4. Discriminant Validity

Discriminant validity was measured along four heterogeneous factors with the possible psychological impact on psychological maladjustment of subfertile women before treatment. We evaluated the relationship between dimensions of psychological maladjustment based on the Hungarian version of SCREENIVF and factors with possible psychological impact—as an indication, compliance to WHO guidelines for physical activity (at least 150 min/week physical activity before the treatment), concurrent reproductive trauma (defined as coping not only with the condition of infertility, but with previous early miscarriage as well), and the type of treatment (type of the treatment has been divided into three categories: Diagnostic testing, Pre-ART and ART). A significant difference was found concerning previous miscarriage in the helplessness cognitions dimension between patients (*p* = 0.018). SCREENIVF was invariant across other risk factors (Table 6).

The differences between social support (SSQ), depression (BDI-13), and fertility-related quality of life (FertiQoL) between women classified as at risk and not at risk were evaluated using Mann–Whitney U-test. The original scoring suggests using an at-risk score when one or more of the five dimensions show a clinically relevant problem [32]. The current analysis calculates a cut-off at two or more dimensions showing a clinical discrepancy. In this case, all the studied scores and sub-scores on social support, depression, and fertility-related QoL showed a significant difference when patients were divided by the presence of risk (Table 7).

## 4. Discussion

The negative effects of infertility on emotional-, social- and somatic well-being is well known and based i. a. on unfulfilled child-wish, the presence of reproductive disorder, and its treatment [60]. Before the treatment, stigmatizing hirsutism or acne in Polycystic Ovary Syndrome (PCOS) or severe pelvic pain and dyspareunia in endometriosis are examples of the somatic and emotional effects caused by the underlying disorder. However, effects of the intense treatment, such as ovarian hyperstimulation, waiting for pregnancy test results, or treatment failure, could also be exhausting [61]. ART is considered a multidimensional stressor; the treatment is a primary stressor, and the unpredictable outcome is another major stressor [62].

Psychosocial screening of patients at the start of ART treatment to provide tailored care is of considerable importance but not yet feasible in daily routine care in Hungary. Therefore, the purpose of this study was the validation and cultural adaptation of the SCREENIVF tool. We aimed to develop a Hungarian version equivalent to the original English version, culturally adapt it to the target population, and assess its psychological properties in a cross-sectional study in Hungary.

The Hungarian version confirmatory factor analysis indicated a good fit. All dimensions were reliable (α ≥ 0.70). Cronbach’s alpha of all dimensions was excellent and varied from 0.825 to 0.904. Strong correlations were found between the total scale of FertiQoL and anxiety (R = −0.507, *p* < 0.001), depression-(R = 0.554, *p* < 0.001 and helplessness cognitions (R = −0.747, *p* < 0.001) and moderate correlation with acceptance cognitions (R = 0.317, *p* = 0.015). Based on our study, it could be claimed that the SCREENIVF tool is valid and reliable for measuring psychological maladjustment before starting ART treatments. The Hungarian version of SCREENIVF showed moderate validity with correlation coefficients.

In a multicenter prospective study, Smeenk et al. demonstrated with survey methods that pre-treatment levels of perceived anxiety (*p* = 0.01) and depression (*p* = 0.03) are in close correlation with outcome measures of IVF and ICSI. Infertility and the perceived lack of control by patients during ART treatment make the situation a long-term stressor. The treatment, particularly a failed one, could be emotionally and physically demanding [18,20], increasing perceived distress, anxiety, and depression [22].

Nonetheless, contrary to single studies based on meta-analyses, no compelling evidence exists that emotional distress (anxiety, depression) affects the outcome of ART. Boivin et al. revealed in a meta-analysis of prospective psychosocial studies on emotional distress in infertile women that pre-treatment emotional distress caused by fertility problems or other life events co-occurring with treatment will not compromise the chance of achieving pregnancy [63]. 

Fifteen years ago, Verhaak et al. drew attention to the emotional aspects of IVF as well and reviewed 25 years of empirical research on different phases in the treatment process, the various aspects of emotional response (anxiety, depression, or general distress), and specific risk and protective factors such as personality characteristics, coping skills, cognitions concerning fertility problems and social support, but also not yielded compelling evidence for significant negative emotional consequences of unsuccessful treatment. Authors unexpectedly found a lack of enhanced anxiety and depression levels in women starting fertility treatment despite the history of several years of fertility problems, which was explained by the fact that IVF may be the treatment as the first step toward a solution to their fertility problems. Based on the review, most women seem to be able to deal effectively with the burden of successive, even unsuccessful, cycles and adjust well. However, due to their considerable number, psychological support should target women at risk of developing adjustment problems following failed cycles [64].

Although the patients’ perception is important, they are at risk of psychological maladjustment even if they do not reach the level of clinical pathology before or during the cycles. However, the level of maladjustment also depends on the specificity and sensitivity of the measurement tool. Smenk et al. reported 5.6 (SD = 5.1) for the BDI-13 score investigating 291 women who reached embryo transfer, which shows great similarity to our result [4.84 (SD = 4.88)] and refers to a normal mood state. It is suggested that concerning patients with fertility problems, fertility-specific scales are suitable for setting up more sophisticated analyses than standardized generic instruments [26,27,28,29,47,65,66,67]. Generic scales cannot easily detect differences between infertile women and their partners, as they do not represent all the unique problems experienced by infertile patients [32,68,69].

This more specific approach also necessitated the development of SCREENIVF. In the Netherlands, SCREENIVF is used in the daily clinical routine and underpins the feasibility of tailored psychosocial care for clients. The development and validation of the tool were based on three high-quality studies. In the first study, Verhaak et al. investigated the psychometric characteristics of a screening tool, SCREENIVF, to identify women at risk of emotional problems at an early stage of treatment. Two hundred seventy-nine women in their first IVF cycle completed SCREENIVF both before the treatment and after the pregnancy test. 34% of the patients were identified as at risk, and 75% of the patients as at risk or not at risk were identified successfully with high (89%) negative predictive [32] (PVN) but low (48% in the total sample and 56% after unsuccessful treatment) positive predictive value (PVP).

In our study, it was a consensus between authors to use FertiQoL for the comparative analysis. Huppelschotten and co-authors measured the difference between the quality of life and emotional status of infertile women and their partners using the same measurement tools in the second Dutch cross-sectional study [69]. Women self-reported lower QoL in emotional [52.5 (0.0–100.0)], mind-body [66.7 (0.0–100.0)] and social [75.0 (4.2–100.0)] domains of FertiQoL [total score 70.8 (14.6–96.9)] and significantly higher scores by SCREENIVF (63.8% at risk) than their partners (45.6%), indicating higher exposure on risk for developing emotional maladjustment. Risk by dimensions varied between 9.9% (Depression) and 34% (Helplessness). The authors found similar values using FertiQoL by women, the median of the emotional scale [52.5 (0.0–100.0)] was the lowest, and the value of the relational scale was the highest [79.2 (2.5–100.0)], as by the Hungarian female sample [54.17 (IQRL 44.79, IQRU 71.88), 79.17 (IQRL 69.79, 91.67 IQRU) respectively].

Ockhuijsen et al. examined the construct and criterion validity of the original Dutch version of SCREENIVF in the third perspective, longitudinal RCT among both genders (468 women and 383 men) undergoing fertility treatment and compared the tool to the Hospital Anxiety and Depression Scale (HADS). They found a good fit for the factor model. For women, 68.7% of the variance was explained by the sum score scale of SCREENIVF by time 1 (before treatment). They found good construct validity, but the predictive validity was poorer than the concurrent validity, and the authors caution against using it as a predictive tool [2].

Reviewing the above study, Boivin also draws our attention [22] to the fact that the cross-sectional prediction (concurrent validity) of SCREENIVF to the Hospital Anxiety and Depression Scale (HADS) at baseline was better than the prospective prediction to the HADS on day 10 after embryo transfer during the waiting period for the pregnancy test or six weeks after embryo transfer [2]. In our study, a cross-sectional design was used, and a fertility-specific scale, the FertiQoL, was applied for concurrent validity, which is one of the most frequently used scales to examine psychometric properties of infertility-specific patient-reported outcomes [22] besides the Fertility Problem Inventory (FPI) [66] and Copenhagen Multicentre Psychosocial Infertility-fertility Problem Stress Scale (COMPI-FPSS) [70]. There was already research experience using FertiQoL [26,30], FPI [27,29], and COMPI-FPSS [67] in the Hungarian sample.

In our studied sample, women and their partners dealt with fertility problems on average for six years (59.0 ± 38.4 months). Infertility and the perceived lack of control by clients during ART make the situation a long-term stressor, but a failed treatment could—depending on personality factors—lead to pathologic emotional response, increasing anxiety, and depression, as proved by the vulnerability model of Verhaak et al. [34]. We examined some factors with possible psychological impact concerning the dimensions of the Hungarian version of SCREENIVF. We found a significant relationship between previous miscarriage and the cognition of helplessness (*p* = 0.018). However, no conclusion could be drawn regarding the type of indication or fulfilling the recommended amount of physical activity (150 min/week) before the treatment. Verhaak et al. also found a significant increase in Helplessness (R = 0.22, *p* < 0.01) and a decrease in Acceptance (R = −0.21, *p* < 0.01) of infertility-related cognitions after failed treatment. 

To the best of the authors’ knowledge, after the original Dutch version, English 2.0 [2,35], Persian [71], Portuguese [72], and Turkish [73] version of the tool is already available and validated, and the adaptation of the Polish version is underway [25].

Psychometric properties of the Portuguese version of SCREENIVF were investigated by Lopes et al. [72] The confirmatory factor analysis for the Portuguese version indicated good fit [χ(2) = 188.50, *p* < 0.001; comparative fit index = 0.97; root-mean-square error of approximation = 0.06 (90% CI 0.05–0.07)] and all dimensions were reliable (α ≥ 0.70, except depression for men: α = 0.66). 52% of women and 30% of men were affected by the risk for maladjustment.

One of the most important reasons for withdrawal from ART treatment is the psychological burden on couples [74]. The risk for psychological maladjustment measured with SCREENIVF before treatment and intention to comply with the treatment is assumed to be in association [2]. In a study by Lopes et al., women and men at risk and not at risk for maladjustment reported similar intentions to comply with treatment (*p* > 0.05). For patients with lower helplessness cognitions, higher anxiety was associated with lower compliance intentions (β = −0.45, *p* = 0.01). For patients with higher helplessness cognitions, higher depression was associated with lower intention to comply (β = −0.33, *p* = 0.02), but not for patients with lower helplessness cognitions (β = 0.19, *p* = 0.30). Although the Portuguese study also investigated men’s responses, the Portuguese version was evaluated as a valid and reliable tool for women undergoing any type of fertility treatment [72].

In our study, individuals at risk were classified at first following the screening concept of Verhaak et al. [9], using the original cut-off points. The at-risk score was calculated when one or more of the five dimensions showed a clinically relevant problem and exceeded the cut-off. In a study by Verhaak et al., 34% of the patients were diagnosed at risk, and scores varied between 10–16% per dimension. Anxiety was at 10%, with a mean of 17.3 (4.8). In Huppelschotten et al. [69], 63.8% of the women were at risk, of which 27.4% related to Anxiety. In the Portuguese study [72], 52% of women were affected by the risk for maladjustment.

The ratio of respondents at risk for the original cut-off values was the highest (56.9%) in the dimension of Anxiety. Probably cut-off value overestimates the risk of Anxiety in the Hungarian population. This could have resulted when using the original concept “one or more of the five dimensions of SCREENIVF shows a clinically relevant problem,” which means being at risk. Almost all patients were categorized at risk. Based on such a consideration, we have decided that using two or more dimensions indicates clinically relevant risk; in this case, 50.00% of the women were at risk. Although a relevant cut of the value of Anxiety should be investigated in a further study, this short version (5 + 5 items) of STAI is not validated in Hungarian to have a normal value.

We had also an unexpected result, fewer associations by the Social Support dimension of SCREENIVF were found as it was expected: significant correlations have been found only with SSQ (R = 0.306, *p* = 0.019), BDI-13 (R = −0.468, *p* ≤ 0.001) and relational subscale of FertiQoL (R = 0.341, *p* = 0.009) and against the assumptions no correlation could be described with the Social Scale of FertiQoL (R = 0.192, *p* = 0.149). The lack of correlation could be justified by the difference between the focus of the questions. Questions of the Social Scale in FertiQoL cover social support aspects, social inclusion, and expectations [47].

In a validation study of the Persian version of SCREENIVF, the authors justified the importance of screening prior to the treatment with the fact that 23–30% of couples prematurely quit ART because of the high physical- and emotional burden of therapy. Authors applied not only quantitative methods (content and construct validity), but qualitative methods (face validity) 20 infertile couples and 14 experts examined the items for suitability, difficulty level, relevance, and ambiguity. They found that despite minor cultural differences, SCREENIVF, with the original item structure, has acceptable credibility and reliability and is easy to use for screening for emotional risk factors and designing appropriate interventions for couples under treatment in infertility clinics in Iran [71]. 

The validation study of the Turkish version was conducted among infertile women (mean age 30.6 ± 3.4 years) against the COMPI-FPSS (F = 161.281, *p* < 0.001, multiple explanatory coefficients 77.2%). The authors removed six items with an insufficient contribution. The final scale consisted of five subscales and 28 items. The item-total score correlation coefficients and subscale-total score correlation coefficients obtained for each subscale ranged between 0.31–0.98. The Cronbach’s alpha coefficient was accepted to be reliable (0.77). The ratio of women exposed to risk was considered relatively low; 16% of the participants were above the cut-off scores concerning anxiety and depression, 13.9% in acceptance, 8.2% in hopelessness, and 2% in social support subscales [73]. 

In line with ESHRE guideline on ‘‘Routine psychosocial care in infertility and medically assisted reproduction” [24], the aim of the study, in addition to the validation of the tool, was to advance the complex fertility care approach and foster the development of preventative and tailored support in Hungary. We strongly believe that taking care of behavioral (lifestyle, exercise, nutrition, and compliance), relational (relationship with a partner if there is one, family friends and a larger network, and work), emotional (well-being, e.g., anxiety, depression, quality of life) and cognitive (treatment concerns and knowledge) needs could influence the perceptions, quality of life, well-being, compliance of ART patients.

### Limitations 

Although the Hungarian results confirmed that SCREENIVF is a valid and reliable tool for assessing psychological maladjustment in infertile Hungarian women, it should be borne in mind that the study’s limitations include the sample’s non-representative and non-randomized nature. Respondents were carefully selected to avoid potential confounders, but it made the study population modest. Although they tend to be less affected, ART also has a psychological impact on men. In our study, only women and not couples were investigated. Assessing test-retest reliability would further strengthen the results, although re-administration of the query was not possible. Thus, a subsequent study should also complement testing the invariance across genders and assessing the test-retest reliability. 

## 5. Conclusions

The Hungarian version of SCREENIVF showed moderate validity with correlation coefficients like other European studies. Based on our study’s results, the SCREENIVF tool is a valid and reliable questionnaire to measure psychological maladjustment before the start of ART treatments.

## Figures and Tables

**Table 1:** General characteristics of the sample (*n* = 60).

General Characteristics			
**Socio-demographic data**		**Type of treatment**	
Age (years)	34.6 (±5.2)	IVF/ICSI	50 (82.3)
Education		IUI	1 (2.1)
Low	6 (10.0%)	OI	2 (4.2)
Intermediate	18 (30.0%)	HSG	2 (4.2)
High	36 (60.0%)	Diagnostic testing	5 (31.1)
**Marital status**		**Previous fertility treatments**	
Married	47 (78.3%)	0	26 (43.3%)
Partner	13 (21.7%)	1	16 (26.7%)
Residence		2	12 (20.0)
Urban	45 (75.0%)	3	6 (10.0%)
Rural	15 (25.0%)	**Health Status and Lifestyle**	
Income		BMI (kg/m^2^)	
Low	2 (3.3%)	Mean (SD)	24.2 (±4.9)
Medium	34 (56.6%)	**Self-Rated Health**	
High	24 (40.0%)	Poor	0
**Fertility**		Fair	1 (1.7%)
Relationship (years)		Neither good nor bad	13 (21.7%)
Mean (SD)	7.6 ± 3.8	Good	37 (61.7%)
Child-wish (months)		Excellent	9 (15.0%)
Mean (SD)	59.0 ± 38.4	**Healthy Diet**	
**Parity**		Pay attention	56 (93.3%)
Nulliparous	53 (88.3)	Not really/No attention	4 (6.7%)
Multiparous	7 (11.7)	Tobacco Use	
**Indication (self-report) *n* (%)**		Occasional	2 (3.3%)
Female	26 (43.3)	Non-Smoker	58 (96.7%)
Male	4 (6.7)	**Exercise**	
Dual	14 (23.3)	Regular (4–7 days/week)	6 (10.0%)
Undefined	13 (21.7)	Regular (1–3 days/week)	24 (40.0%)
Diagnostic testing	3 (5.0)	Less often/Not	30 (50.0)

BMI: Body Mass Index, ICSI: Intracytoplasmic Sperm Injection, IUI: Intrauterine Insemination, HSG: Hysterosalpingogram, IVF: In-Vitro Fertilization, OI: Ovulation Induction.

**Table 2:** Assessment of psychological maladjustment based on the Hungarian version of SCREENIVF (*n* = 60).

SCREENIVF					
Items derived from	Spielberger State and Trait Anxiety Inventory	Beck Depression Inventory	Inventory of Social Involvement	Illness Cognition Questionnaire	Items derived from
Number of items (34 items)	5 + 5	7	5	6	6
Dimensions	Anxiety	Depression	Social Support	Helplessness cognitions	Acceptance cognitions
Cut-off value	24≤	4≤	15≥	14≤	11≥
Mean	24.50	2.41	16.74	11.45	15.09
SD	3.29	2.58	3.07	4.07	3.90
Above cut-off (%)	56.9 *	24.1 *	62.21	25.9 *	79.3
Below cut-off (%)	44.1	75.9	37.79 *	74.1	20.7 *

* The ratio of individuals at risk concerning the cut-off values of the dimensions.

**Table 3:** Pre-treatment quality of life, mental status, and social support characteristics of women based on FertiQoL (*n* = 60).

FertiQoL					
	Very poor	Poor	Neither poor nor good	Good	Very good
SRH (%)	1.72	3.45	17.24	65.52	12.07
	Very dissatisfied	Dissatisfied	Neither dissatisfied nor satisfied	Satisfied	Very satisfied
QoL (%)	0.00	6.90	15.52	60.34	17.24
**Domains**	**Mean**	**SD**	**Median**	**IQR lower**	**IQR upper**
Emotional Scale *	57.47	18.47	54.17	44.79	71.88
Mind-Body Scale *	58.62	19.27	58.33	45.83	71.88
Relational Scale *	77.59	18.30	79.17	69.79	91.67
Social Scale *	63.58	17.50	62.50	53.13	75.00
Environment Scale **	66.24	21.38	68.75	61.46	79.17
Tolerability Scale **	62.50	26.41	68.75	48.44	81.25
Core Scale	64.31	14.24	63.54	54.17	72.14
Treatment Scale	64.74	21.36	68.75	52.50	80.63
Total Scale	64.65	13.90	65.07	55.15	75.00

FertiQol: Fertility Quality of Life Tool, IQR: Interquartile Range, QoL: Quality of Life, SRH: Self-Rated Health, * Domain subscales of the Core Scale, ** Domains of the Treatment Scale.

**Table 4:** Correlation between the SCREENIVF dimensions.

		Anxiety	Depression	Social Support	Helplessness Cognitions	Acceptance Cognitions
Anxiety	R	-				
	*p*					
Depression	R	0.745 **	-			
	*p*	**<0.001**				
Social Support	R	−0.353 **	−0.374 **	-		
	*p*	**0.007**	**0.004**			
Helplessness cognitions	R	0.621 **	0.697 **	−0.283 *	-	
	*p*	**<0.001**	**<0.001**	**0.031**		
Acceptance cognitions	R	−0.273 *	−0.358 **	0.127	−0.371 **	-
	*p*	**0.038**	**0.006**	0.344	**0.004**	

Spearman’s rho correlation. * *p* ≤ 0.05. ** *p* ≤ 0.005.

**Table 5:** Criterion validity of the SCREENIVF compared to FertiQoL, Caldwell Social Support Questionnaire, and Beck Depression Inventory (BDI-13) based on Spearman’s rank correlation analyses.

			Anxiety	Depression	Social Support	Helplessness Cognitions	Acceptance Cognitions
**Caldwell SSQ**	Social Support	R	−0.405 **	−0.429 **	0.306 *	−0.528 **	0.447 **
		*p*	**0.002**	**0.001**	**0.019**	**<0.001**	**<0.001**
**BDI-13**	Depression	R	0.773 **	0.899 **	−0.468 **	0.750 **	−0.369 **
		*p*	**<0.001**	**<0.001**	**<0.001**	**<0.001**	**0.004**
**FertiQoL**	Emotional Scale	R	−0.497 **	−0.533 **	0.050	−0.717 **	0.287 *
		*p*	**<0.001**	**<0.001**	0.709	**<0.001**	**0.029**
	Mind-Body Scale	R	−0.585 **	−0.607 **	0.229	−0.843 **	0.290 *
		*p*	**<0.001**	**<0.001**	0.083	**<0.001**	**0.027**
	Relational Scale	R	−0.409 **	−0.321 *	0.341 **	−0.364 **	0.183
		*p*	**0.001**	**0.014**	**0.009**	**0.005**	0.169
	Social Scale	R	−0.335 *	−0.310 *	0.192	−0.476 **	0.312 *
		*p*	**0.010**	**0.018**	0.149	**<0.001**	**0.017**
	Environment Scale	R	−0.108	−0.217	0.199	−0.302 *	0.162
		*p*	0.418	0.101	0.134	**0.021**	0.225
	Tolerability Scale	R	−0.305 *	−0.321 *	0.177	−0.527 **	0.264 *
		*p*	**0.020**	**0.014**	0.185	**<0.001**	**0.045**
	Core Scale	R	−0.578 **	−0.618 **	0.206	−0.783 **	0.318 *
		*p*	**<0.001**	**<0.001**	0.122	**<0.001**	**0.015**
	Treatment Scale	R	−0.226	−0.306 *	0.206	−0.437 **	0.220
		*p*	0.088	**0.019**	0.120	**0.001**	0.098
	Total Scale	R	−0.507 **	−0.554 **	0.230	−0.747 **	0.317 *
		*p*	**<0.001**	**<0.001**	0.082	**<0.001**	**0.015**

Spearman’s rho correlation. * *p* ≤ 0.05. ** *p* ≤ 0.005.

**Table 6:** Difference between dimensions of psychological maladjustment based on the Hungarian version of SCREENIVF and factors with possible psychological impact.

SCREENIVF						
Factors		Anxiety	Depression	Social Support	Helplessness Cognitions	Acceptance Cognitions
Indication ^1^ (Female/Not)	Z	−0.757	−1.051	−0.197	−0.990	−0.721
	*p*	0.449	0.293	0.844	0.322	0.471
PA compliance ^#^ (Reached/Not)	Z	−0.169	−0.328	−0.318	−0.185	−0.306
	*p*	0.866	0.743	0.751	0.853	0.759
Miscarriage (Missed ab/Not) ^2^	*p*	0.352	0.252	0.175	0.018 *	0.795
Type of treatment (Diagnosed/Pre-ART/ART) ^3^	*p*	0.737	0.680	0.361	0.799	0.898

^1^ Mann-Whitney U-test, ^2^ Pearson Chi-Square, ^3^ Kruskal Wallis Test. ^#^ 150 min/week physical activity. * *p* ≤ 0.05.

**Table 7:** Differences between women classified as at risk and not at risk regarding social support (Caldwell SSQ), depression (BDI-13), and fertility-related quality of life (FertiQoL).

		At Risk		Not at Risk			
		Mean	SD	Mean	SD	Z	*p*
**Caldwell SSQ**	Social Support	12.62	2.04	13.66	1.59	−2.021 *	**0.043**
**BDI-13**	Depression	7.55	5.30	2.14	2.26	−4.316 **	**<0.001**
**FertiQoL**	Emotional Scale	50.29	17.07	64.66	17.20	−2.891 **	**0.004**
	Mind-Body Scale	50.86	19.49	66.38	15.86	−3.095 **	**0.002**
	Relational Scale	72.41	17.09	82.76	18.29	−2.803 *	**0.005**
	Social Scale	57.76	16.66	69.40	16.60	−2.329 *	**0.020**
	Environment Scale	60.49	21.03	71.98	20.50	−2.233 *	**0.026**
	Tolerability Scale	55.17	27.45	69.83	23.57	−2.189 *	**0.029**
	Core Scale	57.83	13.26	70.80	12.23	−3.416 **	**0.001**
	Treatment Scale	58.36	21.16	71.12	19.91	−2.531 *	**0.011**
	Total Scale	58.18	13.17	71.12	11.53	−3.438 **	**0.001**

Mann-Whitney U-test. At risk = two or more dimensions of SCREENIVF showing a clinical discrepancy. * *p* ≤ 0.05. ** *p* ≤ 0.005.

## Data Availability

The dataset supporting the conclusions of this article is available from the corresponding author on reasonable request in accordance with MDPI Research Data Policies.

## Abbreviations

ART	assisted reproduction treatment/technics
BMI	Body Mass Index
Caldwell SSQ	Caldwell Social Support Questionnaire
CFA	Confirmatory Factor Analysis
CFI	Comparative Fit Index
COMPI-FPSS	Copenhagen Multicentre Psychosocial Infertility-fertility Problem Stress Scale
COSMIN	COnsensus-based Standards for the selection of health Measurement Instruments
CRT	Concurrent Reproductive Trauma
ESHRE	European Society of Human Reproduction and Embryology
FertiQol	Fertility Quality of Life Tool
FPI	Fertility Problem Inventory
HADS	Hospital Anxiety and Depression Scale
HSG	Hysterosalpingogram
ICSI	Intracytoplasmic Sperm Injection
IQR	Inter Quartile Range
IUI	Intrauterine Insemination
IVF	In-Vitro Fertilization
OI	Ovulation Induction
PCOS	Polycystic Ovary Syndrome
PVN	Negative Predictive
PVP	Positive Predictive Value
QoL	Quality of Life
RCT	Randomized Controlled Trial
RMSEA	Root-Mean-Square Error of Approximation
SD	Standard Deviation
TAU	Treatment-As-Usual
